# Non-Canonical Hh Signaling in Cancer—Current Understanding and Future Directions

**DOI:** 10.3390/cancers7030857

**Published:** 2015-08-27

**Authors:** Dongsheng Gu, Jingwu Xie

**Affiliations:** Departments of Pediatrics, Biochemistry and Molecular Biology, Pharmacology and Toxicology, The Wells Center for Pediatrics Research, 1044 W, Walnut Street, Indianapolis, IN 46202, USA; E-Mail: donggu@iu.edu

**Keywords:** hedgehog, non-canonical, smoothened, Gli

## Abstract

As a major regulatory pathway for embryonic development and tissue patterning, hedgehog signaling is not active in most adult tissues, but is reactivated in a number of human cancer types. A major milestone in hedgehog signaling in cancer is the Food and Drug Administration (FDA) approval of a smoothened inhibitor Vismodegib for treatment of basal cell carcinomas. Vismodegib can block ligand-mediated hedgehog signaling, but numerous additional clinical trials have failed to show significant improvements in cancer patients. Amounting evidence indicate that ligand-independent hedgehog signaling plays an essential role in cancer. Ligand-independent hedgehog signaling, also named non-canonical hedgehog signaling, generally is not sensitive to smoothened inhibitors. What we know about non-canonical hedgehog signaling in cancer, and how should we prevent its activation? In this review, we will summarize recent development of non-canonical hedgehog signaling in cancer, and will discuss potential ways to prevent this type of hedgehog signaling.

## 1. Introduction

For a long time, since its discovery in *Drosophila* in 1980, hedgehog (Hh) signaling was regarded as an essential regulator for tissue polarity during development. It was not until 1996, when two independent research groups discovered somatic inactivating *PTCH1* gene mutations in a genetic skin cancer disease, Gorlin syndrome, and subsequent discovery of activating mutations of *smoothened* (*SMO*) in skin cancer basal cell carcinomas [1,2,3]. It is now clear that there are at least four types of human cancer using activated hedgehog signaling to drive tumor development: basal cell carcinoma, medulloblastoma, rhabdomyosarcoma [4,5], and meningiomas [6,7,8]. 

The current understanding for hedgehog signaling activation in these tumors is described as the following: In normal situation, PTCH1 functions to suppress smoothened signaling but this suppressive effect can be relieved when hedgehog ligands bind PTCH1. In these tumors, somatic mutations of *PTCH1* (inactivating) or smoothened (*SMO*) (activating) result in constitutive activation of the Hh pathway. Vismodegib, a specific inhibitor for smoothened, has been approved by FDA to treat locally advanced and metastatic basal cell carcinomas. 

Mice with *Ptch1* knocked out or with expression of oncogenic *SmoM2* develop tumors similar to Gorlin syndrome patients, BCCs and medulloblastomas. These results provide convincing evidence for the driver role of Hh signaling for cancer development. In addition, rhabdomyosarcomas frequently occur in mice after *SmoM2* expression or *Ptch1* knockout [9,10,11,12]. 

In addition to medulloblastomas and rhabdomyosarcomas, there are numerous types of cancer associated with Hh signaling activation. However, in each tumor type, the mechanism underlying the hedgehog signaling activation differs. For example, *Ptch1* knockout under the control myeloid lineage specific cre LysM results in the development of gastrointestinal stromal-like tumors (GIST) [13], supporting a dominant role of activated Hh signaling for GIST development. On the other hand, in a small cell lung cancer mouse model, although oncogenic *SmoM2* expression is not sufficient to drive tumor formation, it increases the tumor number. Conversely, mice with *Smo* knockout have reduced tumor number [14]. In a Barrett’s esophagus model, *Shh* expression in the epithelium of esophagus results in elevated Hh signaling in the stroma [15,16]. The situation in pancreatic cancer is quite different. It has been shown that *SmoM2* has no effects on Kras-induced pancreatic cancer development [17]. Similarly, prostate-specific expression of oncogenic *SmoM2* does not lead to formation of prostate cancer [18]. It needs to be cautious to interpret the negative data because most mouse models only represent part of cancer progression. For example, the promoting effects on tumor metastasis are hard to determine because of lack of reliable and robust cancer metastasis models for most cancer types in mice. 

## 2. Hedgehog Signaling Modes of Action in Cancer

We propose three major roles of Hh signaling for cancer development: tumor driving effect, tumor promoting role, or regulator for residual cancer cells. Numerous studies have demonstrated a tumor-driving role of activated Hh signaling in BCCs, medulloblastomas, rhabdomyosarcoma, GIST and Barrett’s esophagus [9,10,13,15,19,20]. In this situation, dysregulation of Hh signaling alone is able to drive tumor development. Thus, active Hh signaling in those tumors functions as a tumor driver. For small cell lung cancer (SCLC) at least in the mouse model, activated Hh signaling can increases the severity of the tumor but this pathway alone is not capable of driving tumor development [14]. In this situation, Hh signaling is mainly promoting tumor development. In Kras-driven pancreatic cancer, Hh signaling inhibition has no effects on tumor formation but may promote cancer metastasis [21,22,23,24,25,26,27,28]. In other situations, active Hh signaling can regulate cancer initiating cells or the tumor milieu [29,30]. Residual cancer cells are responsible for cancer recurrence after chemotherapy, and there is evidence to indicate that Hh signaling may be responsible for maintaining the residual cell population following therapy. 

As to the hedgehog signaling modes of action, at least three types have been reported in human cancer. First, hedgehog signaling is constitutively activated through somatic gene mutations in genes like *PTCH1*, *smoothened* (*SMO*) or *Su(Fu)* (Mode 1). Furthermore, hedgehog signaling can be activated through over-expression of hedgehog ligand, leading to either paracrine Hh signaling (stromal cells with activated hedgehog signaling) (Mode 2) or autocrine Hh signaling (cancer cells with activated hedgehog signaling) (Mode 3). In addition, Hh signaling may also be activated via non-canonical signaling, through targeting Gli transcriptional factors (transcriptionally or epigenetically) or independent of Gli transcriptional factors (e.g., regulation of cyclin B1 through PTCH1 [31] or regulation of G-protein signaling via SMO) [32]. The best way to monitor Hh signaling activation is to detect expression of putative hedgehog target genes (e.g., *GLI1*, *PTCH1*, *HIP* or *SFRP1*). In addition, Hh signaling may synergize with other signaling pathways, including EGF signaling, to regulate certain target genes [33]. Since there are already several reviews on the first three modes of hedgehog signaling in cancer, our focus will be on the non-canonical signaling mechanisms.

## 3. Non-Canonical Hedgehog Signaling in Cancer

In normal situation, the transcription factor Gli is activated through ligand binding of PTCH1 and activation of SMO and this is the canonical Hh signaling pathway. However, in some situations, the Gli transcription factors can be activated by other molecules/signaling independent of ligand and SMO. This type of Gli activation is called non-canonical Hh signaling. Non-canonical Hh signaling is mainly investigated in the context of malignant diseases. The molecules/signaling pathways that can bypass the ligand-receptor signaling axis to activate Hh signaling pathway include Kras signaling, TGFβ, PI3K, PKC and epigenetic regulators. 

### 3.1. The RAS-RAF-MEK Signaling Axis

Hedgehog signaling plays a functionally important role in the genesis of Pancreatic ductal adenocarcinoma (PDAC) [34,35]. However, in the PDAC mouse model with oncogenic Kras expression, conditional deletion of Smo in ductal cells does not affect *GLI1* expression in cancer cells and had no effects on the multistage development of PDAC tumors [36], indicating that ligand-receptor Hh signaling is dispensable in pancreatic ductal cells for PDAC progression, and *GLI* transcription in the neoplastic ductal cells is regulated through alternative manners. Since Kras mutation presents almost universally in PDA and is one of the earliest genetic alterations, it is reasonable to speculate that the RAS/RAF/MEK signaling plays a vital role in the non-canonical activation of Hh signaling in cancer cells. 

**Figure 1:**
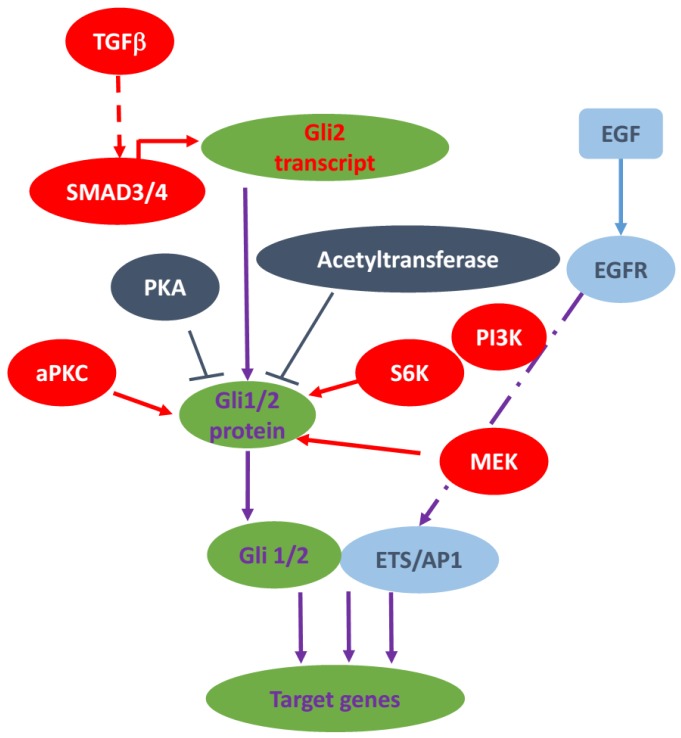
A diagram of typical non-canonical Gli1/2 signaling. There are several ways for regulating Gli transcriptional factors independent of smoothened and Hh ligands. In melanomas, Gli2 transcription is regulated by TGFβ signaling. In esophageal cancer, Gli1 can be phosphorylated by S6K to enhance Gli1 transcriptional activity. In BCCs, a typical PKCi/l can phosphorylate Gli transcription factors to enhance their activities. Previously, it is reported that PI3K and MEK can regulate Gli transcription factors. In addition to the positive regulation, PKA and acetyltransferase are known to negatively regulate Gli protein transcriptional activity. Furthermore, Gli1/2 can also work with downstream effectors of EGF signaling to control transcription of certain target genes.

Indeed, ectopic expression of oncogenic Kras in normal human pancreatic cell line HPDE-c7 can increase transcription activity of Gli molecules [37]. Similar results are also observed after overexpression of mutant Kras in BxPC3 cells, a pancreatic cancer cell line with wild type Kras [36]. Depletion of oncogenic Kras with Kras-targeted siRNAs resulted in a significant down-regulation of the transcription activity of *GLI*, as indicated by *GLI1* and *PTCH1* mRNAs [37] in PDAC cell lines *in vitro*. In gastric cancer cells, the KRAS-MEK-ERK cascade has a positive regulatory role in GLI transcriptional activity [38]. How RAS/RAF/MEK/MAPK cascade causes activation of Gli1 transcription remains to be elucidated. It has been reported that *GLI* transcriptional activity is regulated by the pattern of Gli phosphorylation [39]. In 3T3 cells, if co-transfected with an activated MEK construct, the activity of overexpressed GLI1 can be enhanced by 10 times [40], this enhancement was abolished in an N-terminal deletion mutant of Gli1 lacking the first 130 amino acids [40]; in another report, it was shown that the Ser130 of Gli1 transcription factor are bound and phosphorylated by MAPK cascade [41]. Based on these data, it is reasonable to speculate that the transcription activity of Gli protein could be directly regulated by phosphorylation of the KRAS-MEK-ERK cascade, but this hypothesis need to be confirmed by further study. On the other hand, Gli activity may also be regulated by the KRAS-MEK-ERK cascade by preventing Gli protein degradation. It has been reported that oncogenic Kras blocks proteasome-mediated GLI1 degradation and consequently leads to the activation of Hh signaling in pancreatic cancer cells [37]. In keratinocytes, ERK1/2 activated by EGFR also stabilizes GLI proteins, particularly GLI2, by preventing its degradation via the proteasome pathway [33] (see Figure 1). 

Through the RAS/RAF/MEK cascade, it is possible that the up-stream growth factor receptor tyrosine kinase (RTK) can affect the activity of GLI in a SMO-independent manner or by potentiating the existing Hh Signaling. In human normal keratinocytes, EGFR can transduce signal through RAF/MEK/ERK to cooperate with GLI proteins to selectively regulate some target genes [33], and synergistically induce oncogenic transformation [42]. These findings have been further confirmed in a transgenic BCC mouse model and a subcutaneous xenograft model of pancreatic cancer by genetic and pharmacologic inhibition of EGFR signaling [43], as well as in medulloblastoma [44] and prostate cancer [45]. In NIH3T3 cells, bFGF stimulates *GLI1*-luciferase activity in a MEK-1-dependent manner [40], but in medulloblastoma, FGF is shown as a canonical shh signaling inhibitor in an ERK dependent manner [46], indicating a significant effect of cellular context in the output of signal interaction (see more in Figure 1).

### 3.2. TGFβ Signaling Pathway

TGFβ and its receptors are widely expressed in human body, and its signaling plays a major role in human diseases. During the progression of malignant disorders, TGFβ signaling acts both as a tumor suppressor and as a tumor promoter depending on tumor context (see review [47]). There is increasing evidence to show that TGFβ signal transduction is able to interact with the HH pathway downstream of SMO. Firstly, TGF-β/smad3 cascade was identified as a potent inducer of *GLI2* expression and consequently *GLI1* expression in various cancer cell lines, normal fibroblasts and in keratinocytes [48]. 

Moreover, *GLI2* induction by TGFβ is independent from the Hh/Ptch/Smo axis and de novo protein synthesis [48]. In *SMO*-deleted pancreatic cancer cell line from mouse, TGFβ treatment leads to marked elevation of *GLI1* and *GLI3*, even when *GLI2* expression is undetectable [36]. In a bone metastasis model of breast cancer cell MDA-231, Gli2 in cancer cell induces secretion of PTHrP, an important osteolytic factor, and promotes bone destruction [49]. In this MDA-231 cell line, the *SMO* mRNA is not detectable, and *GLI2* expression was not repressed by SMO inhibitor cyclopamine but regulated by TGFβ signaling (see Figure 1). *GLI2* expression and osteolytic ability of metastatic tumor can be inhibited by abolishing TGFβ signaling, indicating SMO-downstream regulation of *GLI2* via TGFβ cascade [49]. In a series of bladder cancer cell lines, it was found that Hh-independent *GLI2* expression and function contributes to invasiveness. The authors suggested that non-canonical *GLI2* activity may be attributed to RAS and TGF-β, both of these pathways being critical for bladder cancer development [50]. In agreement of these studies, we found TGFβ signaling is critical for Hh signaling-mediated biological functions and carcinogenesis [51]. 

All these studies demonstrated that TGFβ signaling is a regulator of Hh cascade independent of SMO function. To address the mechanism of non-canonical regulation of *GLI* by TGFβ signaling, Dennler and colleagues cloned and analyzed human *GLI2* promoter and identified a 91 bp region harboring SMAD and lymphoid enhancer factor/T cell factor binding sites [52]. This study indicates that in response to TGF-β stimulation, SMAD3 and β-catenin are able to be recruited to the promoter and drive *GLI2* gene transcription. In summary, all these data demonstrate that TGF-β signaling can regulate Gli2 activity by promoting its transcription directly.

### 3.3. PKC Signaling 

Protein kinase C (PKC) is a family of widely distributed phospholipid-dependent serine/threonine kinases, which can be divided into 3 groups: conventional or classic PKC isozymes (cPKCs; α, βI, βII, and γ), novel or non-classic PKC isozymes (nPKCs; δ, ε, η, and θ), and atypical PKC isozymes (aPKCs; ζ, ι, and λ). As a receptor for the tumor-promoting phorbol esters, the function of PKC in cancer has been intensely investigated in the context of cancer [53]. The crosstalk between Hh signaling and PKC signaling has been reviewed [54]. 

Here, we mainly discuss the studies about how PKC signaling activates Gli transcription factors without going through SMO. The role of PKC in Gli activity is controversial. By using LIGHT2 cells, as well as NIH/3T3 cells, Riobo *et al.* found that PMA, a classical PKC and novel PKC stimulator, can induce GLI-luciferase reporter activity in this cell line through PKCδ [40]. This activation can be blocked by the selective MEK-1 inhibitor PD98059 or the dual MEK-1/2 inhibitor U0126, indicating that the PKC/MEK/ERK axis drives the activation of GLI upon PMA stimulation. Neill and colleagues also found that PKCδ promotes *GLI1* expression and PKCα decrease *GLI1* expression in 293 T cells independent of MAPK signaling [55]. Cai and colleagues reported that PKCα increases Gli1 activity via MEK/ERK pathway and PKCδ negatively regulates Gli1 expression through transcriptional down-regulation of *GLI1* mRNA in NIH/3T3 cells and human Hepatoma Hep3B Cells [56]. In mouse BCC cell lines, aPKC-ι/λ was identified as a novel GLI regulator. Although functioning downstream of SMO, aPKC-ι/λ phosphorylates and activates GLI1, resulting in maximal DNA binding and transcriptional activation [57]. In addition, *aPKCι* is also an Hh target gene, and is required for maximal sustained Hh signaling [57]. In summary, the function of PKC signaling the activation of non-canonical Hh signaling is complicated and might be cell type dependent or subtype of PKC dependent (see Figure 1). 

### 3.4. AKT/PI3K Signaling

Riobo and colleagues demonstrated that activation of PI3-kinase/Akt increases Shh-induced Gli transcriptional activity through antagonizing PKA-dependent Gli2 inactivation in several experimental systems [58]. Since loss-of-function mutations in the *PTEN*, a negative regulator of PI3-kinase activity, occur frequently in human cancer and overexpression of IGF I exists in malignant disease, this study indicated that activated AKT/PI3K may facilitate Hh signaling pathway and promote tumor formation. In human *PTEN*-deficient glioblastomas, Hh signaling is activated [59]. Compensatory up-regulation of IGF-1R-PI3K is a candidate for the development of Smo antagonist resistant medulloblastoma, and combination of PI3K inhibitor NVP-BKM120 or the dual PI3K-mTOR inhibitor NVP-BEZ235 with the Smo antagonist markedly delayed the development of resistance [60]. 

In mouse melanoma cells, COS7 cells, human prostate cancer LNCaP cells and human glioma U87 cells, the acquisition of oncogenic RAS-RAF-MEK and/or AKT signaling could be responsible for cell-intrinsic enhancement of GLI1 transcriptional activity driven by enhanced nuclear localization and transcriptional activity [61]. 

### 3.5. TNFα/mTOR Pathway

In a recent study in esophageal adenocarcinomas, it was revealed that GLI1 activity can be enhanced by TNFα/mTOR pathway independent of SMO activity [62]. TNFα induces mTOR activation, and then mTOR phosphorylates its target S6K1. GLI1 protein can be phosphorylated by activated S6K1 at ser84, and this phosphorylation enhances GLI1 function by attenuating SuFu-mediated Gli1 inhibition [62]. This study also confirmed the importance of phosphorylation in the regulation of GLI proteins function.

### 3.6. Epigenetic Regulation of GLI 

Cancer epigenetics is a rapidly developing field of cancer research since more and more epigenetic regulators are found mutated in malignant diseases and more and more evidence is showing the important role of epigenetic dysregulation in cancer growth and metastasis. Not surprising, many compounds have been reported targeting components of the epigenetic machineries for cancer therapy. 

Recently there are some data showing that Hh pathway can be regulated epigenetically during cancer development. SNF5, a tumor suppressor, is core component of the SWI-SNF chromatin remodeling complex. Jagani and coworkers found that SNF5 interacts with GLI1 and is enriched at Gli1-regulated promoter region [63]. Inactivation of SNF5 results in constitutive up-regulation of GLI1 transcriptional activity in malignant rhabdoid tumors cells *in vivo* and *in vitro* [63]. Although detailed mechanisms remain unclear, this study provided a link between abnormal activation of the Hh pathway and deregulated chromatin remodeling in tumor development. 

Multiple endocrine neoplasia type 1 (MEN-1 syndrome), a hereditary condition associated with tumors of the endocrine (hormone producing) glands, and the tumor suppressor gene *MEN1* is frequently mutated in this disease. Gurung and colleagues found that menin combines with Gli1 promoter, and recruits protein arginine methyltransferase 5 (PRMT5) to promote repressive histone arginine methylation, leading to decreased Gli1 expression independent of the canonical GAS1/PTCH1/SMO-mediated Hh signaling [64]. This study revealed a novel non-canonical manner controlling the levels of GLI1 via a menin-mediated epigenetic pathway, and provided a possible mechanism of tumor development of MEN-1 disease, and a rationale for directly inhibiting *GLI1* for treating these tumors. Malatesta *et al.* reported that p300/CREB-binding protein-associated factor (PCAF), a histone acetyltransferase, interacts with GLI1 and is recruited to the promoter of GLI1 responsive gene leading to H3K9 acetylation of Hh target gene promoters and their activation in medulloblastoma and glioblastoma cells, and PCAF downregulation in these cells leads to decreased proliferation and increased apoptosis [65]. However, in hepatocellular carcinoma (HCC) cells, PCAF suppresses Hh signaling by directly acetylating cytoplasmic *GLI1* protein at lysine 518, preventing its nuclear translocation and promoter occupancy [66], whereas deacetylation of Gli1 and Gli2 by histone deacetylases (HDAC) promotes their transcriptional activation in medulloblastoma [67].

BRD4, a conserved member of the bromodomain and extraterminal (BET) family of chromatin readers, plays a critical role in transcription by binding acetylated histones and recruiting transcriptional complexes [68]. Recently, two groups revealed that BRD4 and other BET members can occupy GLI1 and GLI2 promoters directly, and enhance their transcription [69,70]. BRD4 inhibitor JQ1 or I-BET151 treatment inhibits GLI1 and GLI2 expression and growth of hedgehog-driven tumors *in vitro* and *in vivo*, such as basal cell carcinoma, medulloblastoma and atypical teratoid rhabdoid tumor [69,70]. However, the details how BRD4 is regulated and involves in Gli activation, and whether BRD4 facilitates Hh independent non-canonical Gli regulation are needed for further investigation. 

### 3.7. Other Non-Canonical Regulations of Hedgehog Signaling 

Ewing sarcoma family of tumors (ESFT) is characterized by producing an aberrant fusion gene *EWS-FLI1* resulting from the chromosomal translocation t(11;22). EWS-FLI1 can induce *GLI1* transcription by direct binding to the Gli1 promoter, and overexpressed *GLI1* is an important mediator in ESFT development [71]. Earlier study has revealed direct binding of PTCH1 with cyclinB1 in cancer cells, leading to delayed cell cycling [31]. Several studies have indicated signaling of SMO to G-proteins, which is independent of Gli transcription factors [32,72]. How much this G-protein coupling is involved in cancer development is still an unanswered question. 

Gli1 activity is also regulated by energy sensor, AMP-activated protein kinase (AMPK). Recently, Li *et al.* reported that in the situation of insufficient cellular energy, AMPK is activated by accumulated ADP and AMP, and then phosphorylates directly GLI1 at serines 102 and 408 and threonine 1074, leading to suppression of GLI1 transcription activity. In this manner, when energy stores are inadequate, cells or organs slow or postpone developmental steps driven by Hh signaling in order to enhance the chance of survival of a growing animal [73]. In medulloblastoma, this mechanism of Gli1 regulation also exists, and mutation of these three phosphorylation sites may prevent phosphorylation by AMPK and leads to increased GLI1 protein stability, transcriptional activity, and oncogenic ability.

## 4. Conclusions 

It is clear that regulation of Gli transcription factors is not only restricted to the canonical Hh/SMO-dependent cascade but also through the convergence of multiple SMO-independent pathways. These findings not only explain the ineffectiveness and failed clinical trials using SMO antagonists, and also encourage developing new strategies to suppress non-canonical Hh signaling. 

Unlike the canonical pathway, there are different ways by which non-canonical Hh signaling can occur. In some cancer types, more than one type of non-canonical Hh signaling may co-exist. Thus, there is a need of paradigm shift in designing strategies to block hedgehog signaling in cancer. 

First, canonical Hh signaling in cancer is quite limited to tumors with gene mutations in PTCH1 and SMO, which will be sensitive to SMO signaling inhibitors. In most cancer cells, one or more non-canonical Hh signaling modes of action play a critical role in cancer development. Thus, there is an urgent need to understand the specific non-canonical signaling mechanism in a given tumor type before novel mitigation strategies can be established. 

Second, due to the co-existence of activation of multiple signaling pathways in a given cancer types, as well as different mechanisms of non-canonical Hh signaling, combined target therapy will be more effective in suppressing tumor progression. 

Third, despite the existence of multiple aberrant signaling in a given cancer cells, each alteration may affect different stages of cancer development. Thus, physiologically relevant mouse models will be needed to test the role of each aberrant signaling. 

Taken together, we believe that the unsuccessful clinical trials using SMO signaling inhibitors have revealed novel mechanisms underlying Hh signaling activation in cancer, particularly non-canonical Hh signaling. Thus, inhibition of Hh signaling at the level of GLI transcription factors, rather than the level of SMO, might be a more effective way to combat cancer [74,75,76,77]. Our efforts in understanding non-canonical Hh signaling may lead to novel strategies to treat cancer. 
